# The Five-Factor Perceived Shared Mental Model Scale: A Consolidation of Items Across the Contemporary Literature

**DOI:** 10.3389/fpsyg.2021.784200

**Published:** 2022-01-14

**Authors:** Jandre J. van Rensburg, Catarina M. Santos, Simon B. de Jong, Sjir Uitdewilligen

**Affiliations:** ^1^Department of Organisation, Strategy and Entrepreneurship, School of Business and Economics, Maastricht University, Maastricht, Netherlands; ^2^Department of Work and Social Psychology, Faculty of Psychology and Neuroscience, Maastricht University, Maastricht, Netherlands

**Keywords:** perceived mental models, deductive scale development, team cognition, shared mental models, teams

## Abstract

Literature on Shared Mental Models (SMMs) has been burgeoning in recent years and this has provided increasingly detailed insight and evidence into the importance of SMMs within specific contexts. However, because past research predominantly focused on SMM structure as measured by diverse, context-dependent measures, a consolidated multi-dimensional measure of perceived SMMs that can be used across diverse team contexts is currently lacking. Furthermore, different conceptualizations of the dimensionality of SMMs exist, which further impedes the comparison between studies. These key limitations might hinder future development in the SMM literature. We argue that the field of SMMs has now matured enough that it is possible to take a deductive approach and evaluate the prior studies in order to refine the key SMMs dimensions, operationalizations, and measurement. Hence, we take a three-stage approach to consolidate existing literature scale-based measures of SMMs, using four samples. Ultimately, this leads to a 20-item five-dimensional scale (i.e., equipment, execution, interaction, composition, and temporal SMMs) – the Five Factor Perceived Shared Mental Model Scale (5-PSMMS). Our scale provides scholars with a tool which enables the measurement, and comparison, of SMMs across diverse team contexts. It offers practitioners the option to more straightforwardly assess perceived SMMs in their teams, allowing the identification of challenges in their teams and facilitating the design of appropriate interventions.

## Introduction

Effective teamwork is critically important to address the challenges teams face in the course of their work across a wide variety of settings, especially in today’s competitive, demanding, and/or changing work environments (e.g., Kozlowski and Bell, 2013; Mathieu et al., 2019). To effectively face those challenges and perform their tasks, team members have to develop shared mental models (SMMs) – a shared understanding of knowledge among team members relating to important aspects in a team environment (Klimoski and Mohammed, 1994; Mohammed et al., 2010). SMMs are valuable to team members as they reduce uncertainty, lower misunderstandings and conflict, improve coordination and adaptation, and by such means increase effective team functioning (e.g., Cannon-Bowers et al., 1993; Santos et al., 2016; Uitdewilligen et al., 2018). Consequently, SMMs have been shown to be important in the past in many diverse settings and, given the expected challenging (business) world in the years ahead, are likely to remain so in the future (e.g., Mesmer-Magnus et al., 2017; Mohammed et al., 2017; Kniffin et al., 2021).

A thorough scientific understanding of SMMs is thus essential and SMM research has advanced over the years, however, we argue that there are two challenges that need to be resolved in order to drive the SMM field forward. Firstly, a perceived SMM scale to assess and study SMM perceptions in a systematic, consistent, and methodical way across a wide range of contexts is needed. Secondly, to achieve such a scale and work toward a more coherent body of knowledge, the varied conceptualization and underlying dimensions of SMMs need to be consolidated.

To elaborate on the first challenge, SMMs are conceptualized as having both a structural and perceptual component (e.g., Mohammed et al., 2010; Marhefka et al., 2018, 2020). While structure refers to the actual SMM, i.e., existing representations of knowledge held by and shared among team members, perception refers to the awareness of a shared understanding among team members (Mohammed et al., 2010; Marhefka et al., 2020). Researchers have argued that the perception that team members are on the same page is one of the components of a SMM (Klimoski and Mohammed, 1994; Marhefka et al., 2020) and that when team members perceive they have a similar understanding, there is not only a possession of knowledge across individuals but the collective awareness thereof (Burtscher and Oostlander, 2019). Ignorance to the existence of SMMs may imply that teams are unable to leverage their SMMs in working toward collective goals (Rentsch et al., 2009). Despite the importance of analyzing the perception of SMM, research has mainly focused on SMM structure. Much of the literature on SMM structure has been informed by a variety of, often idiosyncratic, measurement approaches that are laborious, time-consuming, and often difficult to apply, as SMMs can be context-specific (Mohammed et al., 2000, 2010; DeChurch and Mesmer-Magnus, 2010a). Mohammed et al. (2000) already observed that the measurement of SMM structure was often inconsistent. Although measurement of SMM structure has become more standardized over the last years, it remains divergent, and context-dependent results are difficult to compare across diverse teams (DeChurch and Mesmer-Magnus, 2010a), leading scholars to suggest that more research is needed on the nature and relevance of general and perceived SMMs (e.g., Mohammed et al., 2010; Marhefka et al., 2018, 2020).

A key factor for such research is the existence of a valid and reliable measure that assesses the perceptions of SMMs in such a way that they fit to a wide range of tasks, teams, and organizational contexts. A general SMM scale would provide a solid anchor to study SMMs in a systematic, consistent, and methodical way across a wide range of contexts and across a much larger spectrum than is feasible with more time-consuming methods. This would furthermore enable SMM structure to be compared to perceived SMM as these different representations of SMMs can have different outcomes in teams (Marhefka et al., 2020). The importance of a perceived SMM scale can also be seen in the fact that there have been a few prior attempts to create or use such a scale (e.g., Levesque et al., 2001; Santos et al., 2015b; Marhefka et al., 2018), but only two such measures were developed and validated following scale development criteria (i.e., Millward and Jeffries, 2001; Johnson et al., 2007). Previous perceived SMM scales faced their own limitations: some combined different dimensions under one factor (e.g., Ellwart et al., 2014; Santos et al., 2015b), did not focus on team members’ cognitive representation of knowledge (e.g., Johnson et al., 2007), or focused only on a few SMMs dimensions (e.g., Millward and Jeffries, 2001; Hsu et al., 2011; Marhefka et al., 2018). Subsequently, a large number of measurement items that are often divergent in content factor structure exist across numerous scales, further convoluting the study of perceived SMMs. To overcome this, a general SMM scale which captures the key theoretical SMM dimensions occurring in the literature is needed.

This brings us to the second issue in the literature, namely the varying perspectives on the dimensional structure of SMMs (Mohammed et al., 2000, 2010, 2021). Mohammed et al. (2021) indicate definitional ambiguity as a limitation in the study of team cognition, including SMMs. Existing theoretical and empirical work offers conflicting ideas regarding the key content dimensions of SMMs. For example, different scholars focus on one or more of the following domains: equipment, task, team interaction, and team (Cannon-Bowers et al., 1993). Others have summarized these in two overarching dimensions of task (merging equipment and task) and team (merging team interaction and team) mental models (e.g., Mathieu et al., 2000). More recently, the conceptualization of SMMs has been expanded to include the temporal domain — a shared understanding of time and other temporal factors which are relevant to the team’s environment (e.g., Mohammed et al., 2015; Santos et al., 2015a). Scholars have observed that it is currently not clear how this dimension relates to the other content domains (Mohammed et al., 2012, 2015). Synthesizing the dimensional structure of SMMs is critical because differences may exist in team members’ representations of each mental model dimension, separate dimensions may be differentially relevant in different contexts, and different dimensions may differently influence various outcomes. Hence, the way in which prior research has theorized and subsequently measured SMMs has differed substantially, and to move forward the dimensionality should be clarified so that future scholars can make more informed theoretical decisions and allow their research to become more comparable.

Addressing these two issues in the contemporary SMM literature is the key goal of the current research. First we will explicitly discuss and theoretically ground the five SMM dimensions. Second if needed, we will refine these conceptualizations of SMMs in order to build a strong overarching theoretical and conceptual framework. Based on that, we then, thirdly, inventory, consolidate, and validate the existing items in the literature into the new Five-Factor Perceived Shared Mental Model Scale (5-PSMMS). This creates a new measure of perceptions of SMMs, which can be applied to teams independent of context. We follow a deductive scale development approach (Hinkin, 1998; DeVellis, 2016), as the current SMM literature is rich in the number and variety of items used, yet lacks an overview and consolidation of these efforts. Based on Hinkin (1998), our approach comprises six steps, namely (1) item inventory, (2) content validity assessment, (3) item reduction, (4) factor structure evaluation, (5) construct validity assessment, and – ultimately – (6) data aggregation assessment to assess the team level properties of our scale with the goal of creating a validated, reliable measure of perceived SMMs.

## Background and Theory of Shared Mental Models

Shared mental models refer to an emergent state – a cognitive construct which is “dynamic in nature and varies as a function of team context, inputs, processes, and outcomes” (Marks et al., 2001, p. 357). As an emergent state, SMMs reciprocally influence not only team outcomes, but also team processes (i.e., team members’ actions that involve interaction with one other and their task environment to translate inputs into outcomes; Marks et al., 2001). Research has shown that when teams develop SMMs, team members organize information and establish response patterns that help them to adapt their behaviors to changes in their environment, and effectively coordinate and perform their work (e.g., Uitdewilligen et al., 2013, 2018, 2021; Santos et al., 2021). Thus, to effectively accomplish their tasks, we argue that it is critical that team members are “on the same page” on *which* tools or equipment to use, *what* tasks to perform, *with whom* they need to interact and coordinate and *how* to do so, and *when* the work has to be accomplished (e.g., Cannon-Bowers et al., 1993; Mohammed et al., 2010, 2017).

### Overview of the Current Measurement of Shared Mental Models

As highlighted above, SMMs are assessed through a wide range of different methodological approaches, yet they are similar in their aim to reveal “the degree of convergence among team members with regard to the content of known elements and the relationships between elements” (Mohammed et al., 2000, p. 129). Researchers study SMMs using different techniques, such as paired comparison ratings (or similarity ratings), rating scales, card sorting, concept mapping, cognitive mapping and content analysis (Mohammed et al., 2000, 2010; DeChurch and Mesmer-Magnus, 2010a). According to Mohammed et al. (2021, p. 480), the measurement of team cognition, and therefore of SMMs, “has been marked by fragmentation,” as can be seen in the numerous measurement approaches and adaptations made to them. In the following sections we elaborate on the most widely used measures of structure and perceptions of SMMs, including their advantages and disadvantages.

#### Measures of Shared Mental Model Structure

##### Paired Comparison Ratings (Or Similarity Ratings)

In paired comparison ratings, participants are presented with a matrix in which concepts related to their tasks are listed along one side and the top, or with a list of pairs of brief statements related to their tasks. These concepts or statements are derived from a detailed task analysis (Mohammed et al., 2010). Participants are asked to evaluate the relationship between each pair of concepts or statements on a numerical (scale format) rating [e.g., “-4 (*negatively related, a high degree of one requires a low degree of the other*) to 4 (*positively related, a high degree of one requires a high degree of the other*)”] (Mathieu et al., 2000, p. 277). Examples can be found in Mathieu et al. (2000) and Lim and Klein (2006).

##### Card Sorting

In card sorting, the researchers provide participants with cards that contain critical incidents. Participants are typically asked to sort these cards into categories (piles) that they find meaningful. Categorizations in this format are subsequently scored and aggregated to form mean team scores for comparison and rating (Mohammed et al., 2010). Examples can be found in Smith-Jentsch et al. (2001, 2008).

##### Concept Mapping

In concept mapping, participants are presented with concepts or statements that they need to arrange in a hierarchical order (Mohammed et al., 2010), or that they need to select and use to build a concept map (e.g., Uitdewilligen et al., 2021). The concepts are typically predetermined and developed based on a detailed task analysis. Examples can be found in Marks et al. (2000) and Uitdewilligen et al. (2021).

##### Cognitive Mapping

In cognitive mapping, participants present their mental model content without using pre-established concepts, statements, or categories (Mohammed et al., 2010). Instead, written or verbal statements from interactions between team members are coded and organized in a manner which illustrates concepts and the relationships between them (e.g., Waller et al., 2004). The concepts are therefore derived from unstructured participant data and compared between team members to develop a picture of the SMM. Examples can be found in Carley (1997) and Van den Bossche et al. (2011).

#### Measures of Shared Mental Model Perception

##### Rating Scales

In rating scales, participants are provided with a series of statements and asked to respond to them using a fixed response format – e.g., from 1 = *strongly disagree* to 7 = *strongly agree*. Items can be worded according to task-related concepts (e.g., Marhefka et al., 2020) but are not necessarily informed by task analyses and can be predetermined based on the theoretical content of SMMs (e.g., Santos et al., 2015b). These measures are aggregated from individual perceptions of mental models to the team level. Examples can be found in Johnson et al. (2007) and Santos et al. (2015b).

### Comparing Measures of Structure and Measures of Perception

A variety of measures of SMMs exists, each with its own unique strengths, but also its problems and limitations. As such, we discuss the advantages and disadvantages related to measures of structure and measures of perceptions of SMMs in the sections below.

#### Measures of Shared Mental Model Structure

##### Advantages

Measures of SMM structure are typically related to a specific task and based on rigorous task analyses, and hence contain specific, in-depth, task-related information. As they are based on task analyses, these measures enable the assessment of SMM similarity (i.e., the degree to which mental models are compatible among team members) and SMM accuracy (i.e., the degree to which mental models correctly represent the team task when compared with a subject matter expert, SME; Edwards et al., 2006). Concept mapping and cognitive mapping have advantages over, for instance, paired comparison ratings. Concept mapping allows participants to create and visualize a concept map and assess the relationship between the main concepts, and hence SMMs reflect procedural or sequential task elements. Cognitive mapping allows participants to determine the content for inclusion in the SMM and researcher influence is therefore limited (DeChurch and Mesmer-Magnus, 2010a; Mohammed et al., 2010).

##### Disadvantages

These measures often differ in the manner and extent to which they assess the structure of SMMs. Representations of mental models across similar teams can therefore differ due to the measure being applied, rather than actual structural differences. This problem is further exacerbated when studying diverse teams that do not share tasks or outcomes, or are dissimilar in terms of team context, such as task interdependence, task complexity, or structure (Kozlowski and Bell, 2013). These techniques are often context-dependent, as they have to be informed by a rigorous and detailed task analysis to identify the concepts or statements that are relevant to a particular team in a particular organization (Mohammed et al., 2010). While this enables the measurement of task-specific content inherent to the team’s SMM, it presents practical difficulties in studying teams in the field as it is a difficult, complex, time-, and resource-consuming task (Mohammed and Hamilton, 2012). There are critical limitations of such custom-made measures, namely that data collection “is time-consuming, is labor intensive, takes longer to analyze (and thus do not render ready feedback for constituencies in the field)” (DeChurch and Mesmer-Magnus, 2010a, p. 10) and results are difficult to compare and integrate across a range of settings.

#### Measures of Shared Mental Model Perception

##### Advantages

Measures of SMM perception provide researchers and practitioners with insight on the awareness of team members of the existence of SMMs, without which they would arguably be unable to capitalize on these models in working toward team goals (Marhefka et al., 2020). Measures of SMM perception require less time and effort from researchers to use in their questionnaires and are easier for participants to assess and can therefore be used with relative ease (DeChurch and Mesmer-Magnus, 2010a; Mohammed et al., 2010). Analyses of perception measures are also simpler to execute and compare within and across samples than conducting content analyses on team interactions or participant-driven representations of SMMs (as is the case in cognitive maps, for example). Participants can complete measures of perceived SMMs within a few minutes, given the relative simplicity of completing questionnaires. These measures are not dependent on tasks and task analyses and the generic item content can be applied in any team context where people work together toward a shared goal. The influence of researchers is limited because the SMM content and dimensions are defined in a manner that can be applied to teams in general and they are therefore free from qualitative interpretations by the researcher (Mohammed et al., 2010). In addition, measures of perception enable replication across a broad spectrum of teams and over time. The comparison between different teams is not reliant on task analyses and therefore simple to code and analyze while completing a series of questionnaires spread over a specific time is easy to coordinate.

##### Disadvantages

The main disadvantage of measures of SMM perceptions is that the nature of rating scales is such that items are not directly compared to one another by the participant and hence offers no indication of the structure of the individual’s mental model. Measures of SMM perceptions may yield false indications of sharedness among team members in cases where these perceptions are inaccurate (Marhefka et al., 2020). As such, it is important to select and prepare respondents well and check the data for outliers and inconsistencies.

### The Need for a Measure of Shared Mental Model Perceptions

Thus, the context dependency typically inherent in measures of SMM structure implies that different teams are likely to develop divergent SMM representations, which are difficult to compare. Currently, no measure offers the potential of comparing actual or perceived SMMs across different teams in a manner that reflects its full factor structure. The lack of a validated and generalizable scale complicates the study and comparison of SMMs across different functions as well as the comparison between the structure and perceptions of SMMs. To overcome this difficulty, it is crucial to have a measure that assesses the perceptions of SMMs that can be completed by team members from various teams and organizational contexts.

Although several scales with many measurement items exist, none of these reflect all the contemporary dimensions of SMMs. The perceptions of SMMs warrant their own measurement as was already acknowledged by Klimoski and Mohammed (1994) when they suggested that SMMs are in part a reflection of team members’ perceptions of their knowledge structures. The awareness of the existence of SMMs is important in capitalizing on shared resources in the team as members may possess consensus regarding their organized understanding and representations of knowledge but be ignorant of this fact (Rentsch et al., 2009). Marhefka et al. (2020) support the notion that perceptions of SMMs are an important aspect of their manifestation in teams and found that perceived SMMs and actual SMMs (i.e., structured representations of the SMM and its content) had different, but complementary team outcomes: perceived SMMs were associated with greater team viability and actual SMMs with greater performance. The benefits of a perceived SMM scale are twofold. First, it relates to an important issue in the SMM literature, namely the relevance of perceptions of SMMs given their implications for team outcomes and the ability of teams to take action based on their actual mental models. Second, such a scale would be simple to implement, analyze, and compare, hence offering insights into perceptions of SMMs not only within specific teams, but also across different teams. It can be distributed to multiple teams, completed without direct observation, and its results are readily interpretable through simple statistical analyses. Naturally, the perceived scale can also be used in comparison, or conjunction, with assessments of actual SMMs. Such a perceived scale would also ease the burden of studying SMMs over time and in dynamic and changing circumstances, given the ease with which it can be distributed and findings can be generalized. Yet, in order to build such a perceived scale, it is vital to adequately capture the dimensionality of SMMs.

### Clarification of the Dimensions of Shared Mental Models

In their foundational conceptualization of SMMs, Cannon-Bowers et al. (1993) identified four different dimensions, namely equipment (i.e., tools and equipment teams use in performing their work), task (i.e., the nature of the tasks teams are required to perform), team interaction (i.e., the way in which teams are structured and team members interact with each other), and team (i.e., the attributes and knowledge team members possess). Cannon-Bowers et al. (1993) illustrate four discrepant (yet related) factors underlying SMMs in teams that inform the team’s core understanding of their function and operation. Drawing on the idea that teams need to perform activities related to the task, and work well together as a team, Mathieu et al. (2000) merged these four dimensions into two: the task (reflecting the equipment and task dimensions) and the team (reflecting the team interaction and team dimensions). The task and team domains are commonly used in literature (Mesmer-Magnus et al., 2017) and, while these domains are effective at describing broad features of a team to which the underlying factors may relate, it is difficult to reconcile that the four factors of Cannon-Bowers et al. (1993) can be paired off into single factors, for instance, that the notion of how members should communicate with one another (interaction) forms part of the same construct as the location of particular expertise in the team (team). The aforementioned factors are likely related, but describe discrepant features of the team’s environment. Although past research has most commonly focused on task and/or team mental models (e.g., Mathieu et al., 2005; Lim and Klein, 2006), this dimensional structure has often not been explicitly theorized, nor tested, and has largely remained an (implicit) assumption. It is therefore critical to consider the four dimensions of SMMs and their unique aspects in order to clarify the nature of the knowledge that is shared among team members.

Although this four-dimensional view captures most of the prior work on SMMs, it does not yet incorporate a key recent development, namely the increasing theoretical and empirical insight that time is a crucial element of a team’s relevant environment (e.g., Roe, 2008; Mohammed and Harrison, 2013). This realization has recently led to the conceptualization of temporal mental models (Mohammed et al., 2015; Santos et al., 2015a). Temporal mental models reflect team members’ shared understanding about how tasks and task components should be sequenced, the pace at which activities in the team need to be completed, and the deadlines and time-related milestones relevant to task accomplishment (Mohammed et al., 2015; Santos et al., 2015a). The research indicates that teams that develop temporal mental models achieve high team performance (Mohammed et al., 2015; Santos et al., 2015a; Marhefka et al., 2018). However, the temporal dimension has not yet been subjected to the same degree of scrutiny as the other four dimensions of SMMs, leaving it vulnerable to (implicit) assumptions about its uniqueness as well as its interrelatedness.

Therefore, a systematic analysis of the existing literature is needed to understand how the dimensions of SMMs can be coherently operationalized and related to one another. We synthesize the full four-factor model of SMMs as originally developed (Cannon-Bowers et al., 1993) with the recently introduced temporal dimension (Mohammed et al., 2015) to conceptualize SMMs as comprising five unique dimensions (as presented in Table 1) representing the most complete, generally applicable factor structure as per current literature. While it is feasible that teams may possess SMMs relating to other aspects of their environment, these five core dimensions capture team members’ understanding of *which* tools or equipment to use, *what* tasks to perform, *with whom* they need to interact and coordinate, and *how* to do so, and *when* the work has to be accomplished. Due to the interactive effects in team functioning, we expect these five dimensions to be strongly correlated to one another as any one dimension can be relevant to one of the remaining four. As an example, in effectively communicating in a team it is important to understand both how (i.e., interaction) and when (temporal) to do so. For the sake of clarity, we slightly rename the dimensions of Cannon-Bowers et al. (1993) to distinguish between *equipment* (e.g., tools and technology), *execution* (e.g., procedures, strategies and contingency plans), *interaction* (e.g., team members’ responsibilities and communication patterns) and *composition* (e.g., team members’ preferences, skills, and habits) mental models, and add to that *temporal* mental models (e.g., Santos et al., 2015a).

**TABLE 1:** Conceptualization of the five SMMs dimensions.

Dimension	Conceptualization
Equipment	Shared understanding among team members about equipment, tools and technology they use in performing their tasks.
Execution	Shared understanding among team members about their duties, work processes and procedures relevant in performing their tasks.
Interaction	Shared understanding among team members about the most appropriate ways and means by which members interact and communicate with one another.
Composition	Shared understanding among team members about members’ knowledge, skills, and abilities.
Temporal	Shared understanding among team members about time and other temporal aspects relating to their environment.

## Hypotheses

### Dimensionality

Our scale represents five main dimensions of SMMs used in the literature that capture team members’ understanding about the equipment, execution, interaction, composition, and temporal aspects of work. Therefore, it is important to confirm the factor structure of our measure, in particular because previous measures have not been developed to represent all these dimensions of SMMs. Thus, we argue that:

*Hypothesis 1: The 5-PSMMS is multidimensional, reflecting a five-factor model representing equipment, execution, interaction, composition and temporal mental model similarity and providing good fit to the data across samples.*

### Convergent Validity

It is important to distinguish our scale from similar and dissimilar related constructs within its nomological network, providing evidence for its construct validity (Hinkin, 1998). We test convergent, discriminant, nomological, and criterion validity (Hair et al., 2018). To test for convergent validity, we assess if there is a positive correlation between our 5-PSMMS with an existing SMM scale which covers four SMM dimensions with a single item per dimension, serving as a fair, albeit limited, measure for assessing general SMMs (Santos et al., 2015b).

*Hypothesis 2a: The 5-PSMMS is positively correlated with the SMM scale of Santos et al. (2015b).*

*Hypothesis 2b: The 5-PSMMS is more strongly correlated with the SMM scale of Santos et al. (2015b) than with other variables in the nomological network.*

### Discriminant Validity

To test for discriminant validity, we compare the 5-PSMMS with transactive memory systems (TMS), as both TMS and SMMs are shared cognitive constructs categorized as emergent states (DeChurch and Mesmer-Magnus, 2010b). TMS refer to a cognitive structure regarding team members’ expertise as well as the shared perception of who knows what (Lewis, 2003; Mesmer-Magnus et al., 2017). By developing TMS, team members can locate and access specific knowledge relevant to team functioning (Lewis, 2003). SMMs are related to TMS and both promote team performance as team members can draw on SMMs and TMS to inform their decisions and adapt their behaviors (e.g., Mesmer-Magnus et al., 2017; Uitdewilligen et al., 2018; Bachrach et al., 2019). SMMs and TMS are distinct, as SMMs refer to the knowledge that is shared among team members, and TMS refer to the location of and access to specialized knowledge that members possess (Lewis, 2003; Mesmer-Magnus et al., 2017).

*Hypothesis 3:* The 5-PSMMS and its dimensions correlate positively with TMS.

### Nomological Validity With Positively Associated Constructs

To further assess our scale’s validity, we will assess the nomological network and also compare our scale to five other constructs, three positively associated with SMMs and, in the next section, two negatively associated constructs (cf. Paulin and Griffin, 2017; Thomas and Lucas, 2019). We start with team learning, which refers to an “ongoing process of reflection and action, characterized by asking questions, seeking feedback, experimenting, reflecting on results, and discussing errors or unexpected outcomes of actions” (Edmondson, 1999, p. 353). It leads to increased knowledge, skills, and abilities within the team which facilitate performance. The construction of meaning and shared understanding that can result from team learning behaviors have been shown to enable the development of SMMs (Van den Bossche et al., 2011). Team learning is related to, but conceptually and empirically different from, SMMs in that it represents the development process of mental representations and structures relating to the team environment rather than those structures themselves.

Next, we assess team reflexivity, a process of overt reflection, analysis and discussion of teams’ performance and strategies for improvement in terms of accomplishments and goals (West, 1996; Tesler et al., 2018). Research has shown that team reflexivity leads to positive team outcomes such as improved team innovation, performance, and learning (Schippers et al., 2015). Team members engaging in team reflexivity actively share information and integrate the product of these discussions in their shared conceptualization of the structure and content of their environment, leading to greater similarity in SMMs (Gurtner et al., 2007; Tesler et al., 2018). Team reflexivity therefore enables the development of SMMs, but is conceptually and empirically different from SMMs as it refers to the processing and interpretation of information within the team, rather than the shared understanding thereof.

Teams often possess a degree of intrateam interdependence, where members are reliant on one another to perform their tasks and achieve their goals, to a greater or lesser extent (Van der Vegt and Janssen, 2003). Interdependence in teams has also been shown to influence knowledge sharing and learning (e.g., Van der Vegt et al., 2010), which is central to the development and emergence of SMMs (Staples and Webster, 2008; Van den Bossche et al., 2011). Maynard and Gilson (2014) propose that interdependence is related to SMMs, and interacts in yielding team outcomes. Van der Vegt and Van de Vliert (2005) also show that interdependence is relevant to perceptions of skill dissimilarity and can therefore be related a shared understanding of which team members possess what skills.

*Hypothesis 4: The 5-PSMMS and its dimensions are positively correlated with (a) team learning, (b) team reflexivity, and (c) interdependence.*

### Nomological Validity With Negatively Associated Constructs

We also assess factors that have been theoretically and empirically negatively associated with SMMs, namely team cognitive diversity and intrateam conflict. Individual and task-related differences between team members can influence their perception of one another, but also have the potential to affect how they develop a shared understanding of important aspects of their work (Lim and Klein, 2006; Mohammed et al., 2010). Team cognitive diversity is the extent of interpersonal differences between team members in terms of their knowledge, skills, values, assumptions, and beliefs (Van der Vegt and Janssen, 2003; Schilpzand, 2010). Different members may possess unique knowledge or interpretations regarding their environment (e.g., Martins et al., 2013). This heterogeneity could lead to conflicting individual representations of mental models, as was shown by Schilpzand (2010), hence team cognitive diversity can be expected to be negatively related to SMMs.

Conflict is a dysfunctional team process that can adversely impact team performance and effectiveness (e.g., de Wit et al., 2012). Three types of conflict can occur in teams (Jehn, 1995; Jehn and Mannix, 2001): relationship conflict (interpersonal incompatibilities characterized by negative affectivity between team members), task conflict (differences in ideas and opinions regarding the team’s task), and process conflict (conflict on how tasks will be executed in terms of duties and resources). de Wit et al. (2012) showed that all three conflict types are negatively associated with emergent states in teams, as they impose constraints on team interaction which is essential to their emergence. In particular, previous research has suggested a negative relationship between SMMs and intrateam conflict (Santos and Passos, 2013; Santos et al., 2015b). Previous studies have shown that one type of conflict can lead to another (e.g., Greer et al., 2008) and that the types often load on the same factor (e.g., Polzer et al., 2006), thus we consider conflict as one construct. We therefore hypothesize that:

*Hypothesis 5: The 5-PSMMS and its dimensions are negatively correlated with (a) team cognitive diversity and (b) intrateam conflict.*

## Scale Consolidation Process

To consolidate the scale, we used a deductive approach aimed at balancing factor analytical structure and model fit characteristics with meaningful content. Thereby, we follow Hinkin’s (1998) and DeVellis’ (2016) scale development procedures. As described in the introduction, many theoretical and empirical studies in the field of SMMs have been published which comprise multiple dimensions of SMMs, hence there is sufficient knowledge to inform a deductive approach to scale development. Conforming to Hinkin’s (1998) steps, we develop the scale across three main stages, namely: (1) item development, (2) scale development, and (3) scale evaluation. In these steps, we balanced theoretical and empirical considerations in selecting items, because when “items load as expected then it will require some judgment in deciding which items to retain” (Hinkin, 1998, p. 171). Following DeVellis (2016), we take a conservative approach when reducing items and thus rather retain a surplus (i.e., redundant items) than eliminating potentially useful content too early.

### Stage 1: Item Inventory and Evaluation

We conducted a systematic literature search to identify published SMMs measures that could be used to derive scale items or to inform item development using EBSCOhost, targeting four specific databases: Business Source Premier, ERIC, PsychARTICLES, and Psychology and Behavioral Sciences Collection. We searched for academic papers published in peer-reviewed journals until February 2019 yielding 216 published papers.

#### Criteria for Inclusion

We only considered papers that directly dealt with SMMs as a construct (reducing the pool to 103 results), and measured SMMs with perceived scales (36 results) or similarity ratings (27 results) as these were the only measures likely to provide usable scale items. This left us with a total of 61 papers from which to extract items.

First, we examined the selected papers and listed all items that measured SMMs using scales. The items of four papers could not be obtained, also not after contacting the authors. We also excluded two papers in sports teams, leaving us with 30 papers for consideration, including 332 items. We removed duplicate items (a consequence of researchers using each other’s scales or items), leaving 19 papers and 188 items.

Next, we listed the items we could obtain from papers using similarity ratings, which we split into two categories (we could not obtain the items of seven papers for categorization). The first category referred to items that could be adapted to reflect SMMs in generic team contexts comprising 85 usable items from eight papers. The second category referred to items that could not be adapted to generic team contexts as the researchers either used concepts, and not statements, to assess SMMs, or items which were inseparable from their contextual basis. We excluded 12 papers with 146 items in this category.

In total, we retained 27 papers (19 using scales, eight using similarity ratings) which contained 273 items (188 scale items, 85 similarity rating items).

#### Item Generation

As our aim was to develop a scale that would be applicable in a wide range of tasks, teams, and organizational contexts, we removed all contextual information from the items. Similarity rating items were adapted into a scale format where participants could indicate their level of agreement with the statement. We also split double-barreled items into items measuring only one issue. Consequently, we had a list of 314 items.

We applied the item stem “*Team members have a similar understanding about*…” to all items and deleted exact copies of items following adaptation, using the clarity and conciseness of their wording, and publication date as basis for retaining the best alternative. We screened all items against the criteria for high-quality scale items defined by Hinkin (1998), namely: items should be short and simple, use familiar language, and be consistent in perspective (describe behavior or feelings), address only one issue, avoid bias, and reverse scored items should be carefully worded to avoid misinterpretation. This filtering process yielded 248 items.

#### Content Validity Assessment

To assess the items in terms of their content validity (i.e., the degree to which they are likely to represent the construct they are associated with; Hair et al., 2018) we drew on two stages of review by panels of SMEs in teamwork and organizational behavior. Associated statistics were calculated to determine which items to retain for use in subsequent exploratory factor analyses (EFA).

##### Subject Matter Expert Review: First Stage

In the first stage, we asked a panel of six SMEs (cf. Hardesty and Bearden, 2004; Colquitt et al., 2019) to categorize all the items (randomized per item) according to the five SMMs dimensions. SMEs were presented with definitions of the five dimensions of SMMs and asked to categorize each item under one or more dimensions. They could also categorize the items under “Other” – to check whether another dimension was hidden in the existing item list – or “Unrelated” – to identify misaligned items. SMEs could write suggestions when selecting either option.

##### Subject Matter Expert Review Process: Second Stage

In the second stage of the expert review process, we asked a panel of 18 SMEs (10 established Organizational Behavior scholars and eight Ph.D. students) to rate how well the items associated with each dimension rated the particular dimension they were linked to. We presented reviewers with a definition of each dimension followed by the items categorized under each of the five dimensions as defined. We provided SMEs with Colquitt et al.’s (2019) instructions to rate how well each item measured the dimension using a scale ranging from 1 (*Item does an* ***EXTREMELY BAD*** *job of measuring the **bolded** concept provided above*) to 7 (*Item does an **EXTREMELY GOOD** job of measuring the **bolded** concept provided abov*e).

##### Analysis and Results: Subject Matter Expert Reviews

Following the guidelines of Colquitt et al. (2019) for assessing content validity, we calculated the Coefficient of Substantive Validity (*C*_*sv*_; frequency of aligned and discrepant categorizations^1^) and Proportional Substantive Agreement (*PSA*; proportion of reviewers agreeing on item categorization) based on the first stage expert review. We calculated the Hinkin-Tracey Coefficient (*HTC*; mean item rating divided by the number of anchors) based on the second stage expert review.

Colquitt et al. (2019) provide guidelines for interpreting the three statistics mentioned above (i.e., *C*_*sv*_,*PSA, HTC*) in terms of five categories of content validity strength based on the degree of agreement between raters (i.e., *very strong, strong, moderate, weak*, and *lack of* agreement). For all three statistics we used criteria of Colquitt et al. (2019) for scales not normed to orbital scales as no such measure was included at this point in the process. Items which showed a *lack of* agreement among SMEs under one or more of the three statistics were not considered for further use (one exception was made for an item under equipment which did not meet this requirement for *C*_*sv*_ to allow content breadth). We thus retained 127 items (9 for equipment, 35 for execution, 31 for interaction, 27 for composition, and 25 for temporal) for further evaluation.

Next, we identified if any duplicate items from the first SME review were still retained in this list. We found one such item in the execution and temporal lists and thus removed it, leaving 34 for execution and 24 for temporal. We then evaluated the remaining 125 items following Hinkin’s (1998) criteria for high quality scale items and potential redundancy, opting to include items which were distinct from one another but related to, and representative of, the theoretical dimension (DeVellis, 2016) with the aim to retain eight to twelve items per dimension. This left us with 49 items (nine for equipment and 10 each for the remaining four dimensions).

Next, we evaluated the 49 retained items in terms of their wording and content breadth. Two items under the equipment and execution dimensions respectively were worded in a manner which appeared less related to the dimensions and we included an alternative format item for each. We also added a new item to the interaction dimension given that no item was directly addressing methods of communication within the team, representing a potential gap between the items and theory. This yielded 56 items (11 for equipment, 13 for execution, 11 for interaction, 10 for composition, and 11 for temporal).

### Stage 2: Scale Development

In the next stage of the scale development process, shortlisted items were evaluated by administering them to a sample which was representative of the target population (i.e., adults working in teams). The purpose of this stage was twofold: firstly, to determine whether the theorized factor structure underlying the scale was supported and, secondly, to identify which items were most suitable to measure these factors.

#### Initial Item Reduction

##### Participants

We recruited our first sample of 311 participants using Prolific^2^. After screening responses for quality in terms of obvious bias (e.g., only one response option selected throughout), failure to properly respond to attention controls (see below), and anomalous data (e.g., reported team size of 300) we retained a dataset of 287 participants. Participants reported working in teams comprising 9.12 members on average (*SD* = 6.28), ranging from three to 35 members, predominantly working in the following sectors: healthcare and social assistance (12.9%), finance and insurance (8.7%), government and public administration (8.4%), and retail (7.7%). Of these participants, 47.4% were female and 35.1% identified as leaders in their teams. The average age was 34 years (*SD* = 9.99), ranging from 19 to 65 years, and the average team tenure was 3.58 years (*SD* = 3.82). Participants hailed from 32 different countries, predominantly the United Kingdom (40.8%), the United States (14.6%), Portugal (10.5%), Poland (6.6%), and Canada (4.5%).

##### Measures

The questionnaire contained the 56 SMMs items which were randomized per participant and measured using a 7-point scale ranging from 1 (*strongly disagree*) to 7 (*strongly agree*). To control for response quality, we included two attention control items instructing participants to select a particular response (e.g., “This item is an attention control item and the Disagree option needs to be selected.”).

##### Analysis

Following Hinkin’s (1998) recommendations we conducted a series of exploratory factor analyses (EFA) to determine whether the theorized factor structure was represented in the data and, if so, to reduce the number of items in the scale based on these results. Because of the expected correlation between factors (separate, but related, facets of SMMs) and to avoid the overestimation of factors as can be encountered with principal components analysis, we used principal axis factoring and oblique rotation (Worthington and Whittaker, 2006; Hair et al., 2018).

##### Results

Six factors were extracted (Eigenvalues > 1). The pattern matrix suggested that four of the five dimensions (equipment, interaction, composition, and temporal) possessed items which loaded sufficiently (>0.40) with no problematic cross loadings (>0.35) to proceed with item reduction (Hinkin, 1998). However, items linked to the execution dimension loaded on more than one factor. Three items loaded on the temporal dimension (>0.40) while two loaded on a sixth factor (>0.40). Upon reducing the items for four factors (excluding execution), we found that the majority of the items (*N* = 12) under the execution dimension now loaded on the same factor as those under the temporal dimension. This finding suggested that – based on the existing items from the literature – *Hypothesis 1* could not be supported and that a problem was incurred at the theoretical level. As such we had to undertake an additional step.

#### Clarification of the Temporal Dimension

To determine why the execution items loaded on the same factor as the temporal items, despite being described as unique in the literature, we analyzed the definitions for both dimensions as well as the items associated with each construct. We identified a potential problem in the manner in which the temporal dimension had been previously defined in the literature. This definition was focused on temporal aspects relating to “tasks,” “task components” and “task accomplishments” (e.g., Gevers et al., 2006; Mohammed et al., 2015; Santos et al., 2015a; Marhefka et al., 2018).

We therefore had to challenge the current conceptualization of temporal mental models, as we discovered that it was not theoretically distinct enough from the other dimensions. More specifically, we found that existing temporal mental model items and conceptualizations overlap with the execution dimension as they state that team members have a shared representation of knowledge about how *tasks* and *task components* should be sequenced. Temporal factors such as sequencing, pace, timing, deadlines, and time-related milestones can be applied more broadly than the performance of tasks alone. For instance, the timing of sharing a new piece of information between members can affect the conditions for team functioning. Time can be considered relevant to all aspects of SMMs as team members can have greater or lesser agreement on the relevance of time in terms of their work and team functioning. The temporal dimension should therefore be considered unique and independent, warranting inclusion as a generalized construct. We therefore redefine temporal mental models as “team members’ shared and organized understanding and mental representation of knowledge about time and the temporal aspects (e.g., sequences, pace, deadlines, and timing) related to their work and the key elements of the work environment relevant to the team.”

We revised temporal items to represent the revised definition of temporal mental models using the 27 temporal items originally retained from the literature as a basis. We identified all words or phrases that were linked to the execution dimension and adapted the items to reflect the revised definition for temporal mental models. Two items were duplicated after adaptation and one of each duplicate pair eliminated. The remaining 25 adapted items were subjected to a further round of content validity evaluation where we asked another panel of SMEs from the field of Organizational Behavior (*N* = 8) to rate the degree to which each item measured the adapted definition of the temporal dimension as well as the task dimension (with particular emphasis on the execution dimension), to determine which items would be likely to better represent a unique factor.

We calculated the *HTC* for items of both dimensions and compared these to determine which items were most convergent on the temporal dimension and most divergent from the execution dimension. We retained or eliminated items across three steps, namely we: (1) retained items with a moderate to strong *HTC* score, (2) eliminated items that were relatively similar, and (3) retained items with weak *HTC* scores if these contributed to construct breadth. On this basis we retained 15 temporal items for further EFA.

#### Final Item Reduction

##### Participants

We recruited a second sample of 301 new participants using Prolific. Using the same criteria as the first dataset, our second dataset contained 275 responses. Teams had on average 7.55 members (*SD* = 4.71), predominantly worked in: information services and data processing (9.1%), health care and social assistance (8.4%), finance and insurance (7.6%), manufacturing (6.9%), and software (6.9%). Of the participants, 37.8% were female and 35.6% identified as leaders in their teams. The average age was 33.35 years (*SD* = 8.14) and average team tenure was 3.63 years (*SD* = 3.41). Participants hailed from 29 countries, but were predominantly from the United Kingdom (43.6%), the United States (18.9%), Portugal (8.7%), and Poland (6.5%).

##### Measures

We administered a questionnaire with 60 items (including the 15 revised temporal items and the original 11 for equipment, 13 for execution, 11 for interaction, and 10 for composition). As before, the items were randomized per participant and measured using a 7-point scale ranging from 1 (*strongly disagree*) to 7 (*strongly agree*). We also included the two attention control items.

##### Analysis

We again conducted EFAs following Hinkin’s (1998) recommendations. We reduced items in a stepwise manner over a series of 15 EFA until we were left with four items per dimension. Following Hinkin’s (1998) and DeVellis’ (2016) guidelines, we created a set of six hierarchical rules that would be applied for every EFA in the series to identify items to eliminate. For every EFA we checked the application of the following rule in the numerical order they are presented and subsequently eliminated items accordingly: (1) eliminate items with no factor loading above the absolute minimum of 0.32; (2) eliminate items that have their strongest loading under a different factor than the majority of items from the same content domain; (3) eliminate items with cross loadings greater than 0.32; (4) eliminate items with loadings below the ideal minimum of 0.40 on the expected main dimension; (5) eliminate worst loading items from alternative item pairs (as discussed in “Content Validity Assessment”); (6) eliminate the worst loading item from each dimension (one per dimension per EFA).

##### Results

We used the rules described in the prior section to eliminate items from dimensions, and first focused on the four non-temporal dimensions as the temporal dimension was revised. This yielded a set of seven items per dimension. We then included the temporal items in the analyses and applied criteria (1) to (4) that left us with nine temporal items in addition to the seven items under each other dimension. Four temporal items had similar content (relating to deadlines) and we eliminated two of the four to ensure that the retained items reflected as much of the theoretical content as per the dimension’s definition. This yielded seven items under each of the five dimensions and we reduced this further on the basis of criteria (1) to (6) until we were left with the final set of 20 items (four items under each of the five dimensions). All initial Eigenvalues were around 1 and given our strong theoretical consideration we chose to specify a five-factor extraction (Hair et al., 2018).

The subsequent factor solution reflected five factors with four items attached to each (Table 2), which conforms to Hinkin’s (1998) suggestion of 4–6 items per dimension. Reliabilities for the scales were acceptable (Hinkin, 1998), as all values were above 0.70 (ranging from 0.82 for equipment to 0.88 for composition, with an overall scale reliability of 0.94). Overall, this yielded good support for *Hypothesis* 1 and the 20 items (see Table 3) were consequently deemed adequate for entering the next stage.

**TABLE 2:** Exploratory factor analyses pattern matrix – final item reduction (Sample 2).

Item	Factor				
	1	2	3	4	5
**Equipment**					
How to use other team members’ equipment	*0.02*	*−0.01*	**0.71**	*0.00*	*−0.08*
What equipment is important for which tasks	*−0.01*	*0.02*	**0.72**	*0.02*	*0.11*
The tools needed to complete our tasks	*0.06*	*0.04*	**0.66**	*0.02*	*0.11*
The technology needed to complete our tasks	*0.02*	*−0.07*	**0.66**	*0.01*	*−0.02*

**Execution**					
Specific strategies for completing various tasks	**0.58**	*−0.06*	*0.18*	*0.02*	*−0.04*
How to deal with the task	**0.55**	*0.00*	*0.17*	*0.03*	*0.11*
How best to perform our tasks	**0.64**	*−0.10*	*−0.02*	*0.10*	*0.14*
The relationships between tasks	**0.56**	*−0.10*	*0.02*	*0.08*	*0.07*

**Interaction**					
How to communicate with each other	*−0.05*	*−0.07*	*−0.03*	**0.86**	*−0.03*
Sharing information with each other	*0.20*	*−0.02*	*0.01*	**0.46**	*0.12*
How we should interact with each other	*−0.07*	*−0.19*	*0.12*	**0.60**	*0.04*
The best methods to communicate with each other	*0.10*	*0.10*	*0.02*	**0.81**	*0.03*

**Composition**					
Each other’s knowledge	*−0.03*	**−0.77**	*0.02*	*0.01*	*0.05*
Each other’s abilities	*0.08*	**−0.74**	*0.06*	*0.03*	*−0.02*
Each other’s skills for doing various team tasks	*0.00*	**−0.73**	*−0.03*	*0.01*	*0.21*
Each other’s individual strengths and weaknesses	*0.10*	**−0.71**	*0.03*	*0.08*	*−0.16*

**Temporal**					
Our deadlines	*−0.08*	*−0.04*	*0.12*	*0.11*	**0.63**
How quickly we need to work	*0.19*	*−0.04*	*0.02*	*0.03*	**0.54**
Appropriately timing our work	*0.17*	*0.02*	*0.00*	*0.07*	**0.72**
Coordinating the timing of our work	*0.10*	*−0.22*	*0.13*	*0.00*	**0.51**

Rotation sums of squared loadings*^a^*	6.72	6.16	6.32	6.59	5.40

*All items share the item stem “Team members have a similar understanding about…”; N = 275 individuals; final set of items following item reduction using the second sample of participants.*

*^a^Principal axis factor extraction and direct oblimin rotation.*

**TABLE 3:** 5-PSMMS items.

Dimensions and items	Obtained or adapted from
**Equipment**	
How to use other team members’ equipment	Lim and Klein, 2006
What equipment is important for which tasks	Kang et al., 2006
The tools needed to complete our tasks*^a^*	Santos et al., 2015b
The technology needed to complete our tasks*^a^*	Santos et al., 2015b
**Execution**	
Specific strategies for completing various tasks	Johnson et al., 2007
How to deal with the task	Van den Bossche et al., 2006
How best to perform our tasks	Guchait et al., 2014
The relationships between tasks	Kang et al., 2006
**Interaction**	
How to communicate with each other*^a^*	Lee and Johnson, 2008
Sharing information with each other*^a^*	Johnson et al., 2007
How we should interact with each other*^a^*	Lee and Johnson, 2008
The best methods to communicate with each other*^b^*	N/A
**Composition**	
Each other’s knowledge	Lee and Johnson, 2008
Each other’s abilities*^a^*	Lim and Klein, 2006
Each other’s skills for doing various team tasks*^a^*	Johnson et al., 2007
Each other’s individual strengths and weaknesses	Burtscher and Oostlander, 2019
**Temporal**	
Our deadlines*^a^*	Levesque et al., 2001
How quickly we need to work*^a^*	Marhefka et al., 2018
Appropriately timing our work*^a^*	Mohammed et al., 2015
Coordinating the timing of our work*^a^*	Levesque et al., 2001

*All items share the item stem “Team members have a similar understanding about…”.*

*^a^Items that were adapted from the original.*

*^b^Newly created item.*

### Stage 3: Scale Evaluation

Following item reduction, we evaluated the degree to which items represent the theoretical model. We conducted a third study, where we performed a confirmatory factor analysis (CFA) to test model fit and conducted evaluations of construct validity (Hinkin, 1998; Hair et al., 2018). Then we tested if the measure had distinct team-level constructs (James et al., 1993; Bliese, 2000) by conducting a fourth study using working teams. Finally, we tested whether the criterion related validity of the scale at the team level in a fifth study comprising working teams.

#### Confirmatory Factor Analysis

##### Participants

To assess the fit of the items to the theoretical model, we recruited a third sample of 310 new participants using Prolific. We followed similar procedures as before and obtained a dataset of 288 participants. Participants reported working in teams comprising 7.25 members on average (*SD* = 4.25) predominantly working in the following sectors: information services and data processing (12.85%), government and public administration (9.7%), finance and insurance (8.0%), and software (7.3%). Of the participants, 45.5% were female and 29.5% were leaders. The average age was 33.24 years (*SD* = 8.67) and the average team tenure was 5.52 years (*SD* = 5.32). Participants hailed from 30 countries, predominantly the United Kingdom (43.8%), Poland (10.4%), United States (9.0%), and Portugal (7.3%)^3^.

##### Measures

Our survey had the SMMs items and the measures to assess convergent, discriminant, and nomological network validity. All measures used a 7-point scale.

*Shared Mental Models.* We measured SMMs with our 20 items (α = 0.94) and the existing four-item SMM scale of Santos et al. (2015b; α = 0.81) to assess convergent validity.

*Positively Related Variables.* We measured TMS with the 15-item scale of Lewis (2003; α = 0.84) to test for discriminant validity. Team learning was assessed with the seven-item scale of Edmondson (1999; α = 0.76). Team reflexivity was assessed with the eight-item scale of Carter and West (1998; α = 0.76). Interdependence was assessed with the eight-item scale of Van der Vegt and Janssen (2003; α = 0.80).

*Negatively Related Variables.* Team cognitive diversity was assessed with the four-item scale of Van der Vegt and Janssen (2003; α = 0.64). Intrateam conflict was assessed with the nine-item scale of Jehn and Mannix (2001; α = 0.92).

##### Analysis

We conducted a series of CFAs using AMOS (version 25) to determine the model’s adequacy, including an unconstrained model, a single factor model, and the theorized factor model. We took two steps in order to assess convergent, discriminant, and nomological validity. Firstly, we calculated the construct reliability (CR), average variance explained (AVE), and maximum shared variance (MSV) for the theorized model (Hair et al., 2018). In addition, we analyzed the correlations between our scale and the variables identified for testing convergent, discriminant, and nomological validity (Hair et al., 2018).

##### Results

Table 4 shows the results of the series of CFAs. We obtained acceptable model fit, which supports the structure of the scale and its ability to measure the theorized constructs (Hair et al., 2018). This supports *Hypothesis 1*. The CR values for the five dimensions ranged from 0.80 (equipment) to 0.87 (interaction) as is shown in Table 5, further supporting *Hypothesis* 1.

**TABLE 4:** Confirmatory factor analysis - model fit indices (Sample 3).

Model	χ ^2^	*df*	χ 2/*df*	*p*	CFI	SRMR	RMSEA [90% CI]	PCLOSE
Model 1	3225.91	190	16.98	<0.001	0.00	0.44	0.24 [0.23, 0.24]	0.00
Model 2	757.21	170	4.45	<0.001	0.81	0.07	0.11 [0.10, 0.12]	0.00
Model 3	246.94	160	1.54	<0.001	0.97	0.05	0.04 [0.03, 0.05]	0.84

*Model 1: independent model; Model 2: single factor model; Model 3: five-factor model. CFI, comparative fit index; SRMR, standardized root-mean-square residual; RMSEA, root mean square error of approximation; CI, confidence interval.*

**TABLE 5:** Construct reliability, average variance extracted, and convergent and discriminant validity (Sample 4).

					Correlations				
Dimension	CR	AVE	MSV	MaxR(H)	1	2	3	4	5
(1) Equipment	0.80	0.50	0.61	0.81	*(0.71)*				
(2) Execution	0.81	0.52	0.71	0.81	0.78 ***	*(0.72)*			
(3) Interaction	0.87	0.63	0.56	0.89	0.60 ***	0.75 ***	*(0.80)*		
(4) Composition	0.86	0.61	0.54	0.87	0.64 ***	0.74 ***	0.69 ***	*(0.78)*	
(5) Temporal	0.85	0.58	0.71	0.85	0.63 ***	0.84 ***	0.71 ***	0.65 ***	*(0.76)*

N = 288 individuals. CR, construct reliability; AVE, average variance explained; MSV, maximum shared variance; MaxR(H), maximum reliability. Square root of AVE (H) on diagonal of correlation table in italics and between brackets; CR is computed “from the squared sum of factor loadings for each construct and the sum of the error variance terms for a construct” and >0.70 is sufficient (Hair et al., 2018, p. 676). AVE is calculated as the sum of all standardized factor loadings divided by the number of items in the particular scale and the suggested cut-off value is 0.50. To establish discriminant validity the square root of AVE for a particular factor should be larger than its correlation with other factors (Hair et al., 2018). ***p < 0.001.

Table 6 shows the correlations between the 5-PSMMS with other variables. In support of convergent validity, our scale correlated significantly with the SMMs scale of Santos et al. (2015b), *r* = 0.66, *p* < 0.01. This finding supported *Hypothesis* 2a. The scale also demonstrated that the correlation between the 5-PSMMS and the SMM scale of Santos et al. (2015b) exceeded those between the 5-PSMMS and all other included variables. By converting correlations to Fischer’s *Z* scores we found that the correlation between the 5-PSMMS and the SMM scale of Santos et al. (2015b) was significantly larger than between the 5-PSMMS and all variables in this study at the *p* < 0.01 level, supporting *Hypothesis 2b*. In particular, the scale demonstrated a significant, large correlation (*r* = 0.55, *p* < 0.01; which was smaller than the correlation with the alternative SMM scale) with the TMS scale of Lewis (2003), supporting discriminant validity and lending support to *Hypothesis 3*. We also note significant moderate correlations with factors positively associated with SMMs (i.e., *r* = 0.45 for team learning, *r* = 0.34 for team reflexivity, and *r* = 0.36 for team interdependence; all *p* < 0.01), thus supporting *Hypotheses 4a, 4b*, and *4c*. Two factors negatively associated with SMMs showed significant negative correlations: *r* = –0.24 for team cognitive diversity and *r* = –0.37 for intrateam conflict (both *p* < 0.01), supporting *Hypotheses 5a* and 5*b*.

**TABLE 6:** Descriptive statistics and correlations for convergent and discriminant validity (Sample 4).

Variables	*M*	*SD*	1	2	3	4	5	6	7	8	9	10	11	12
(1) Equipment	5.54	0.88												
(2) Execution	5.31	0.91	0.62 **											
(3) Interaction	5.47	1.02	0.52 **	0.64 **										
(4) Composition	5.42	0.91	0.54 **	0.61 **	0.63 **									
(5) Temporal	5.40	1.01	0.52 **	0.69 **	0.64 **	0.55 **								
(6) 5-PSMMS (current study)	5.43	0.78	0.77 **	0.86 **	0.85 **	0.81 **	0.83 **							
(7) SMM scale (Santos et al., 2015b)	5.52	0.82	0.54 **	0.52 **	0.54 **	0.62 **	0.51 **	0.66 **						
(8) Transactive memory systems	5.48	0.61	0.36 **	0.45 **	0.49 **	0.49 **	0.47 **	0.55 **	0.61 **					
(9) Team learning	4.77	0.92	0.29 **	0.43 **	0.43 **	0.29 **	0.38 **	0.45 **	0.45 **	0.53 **				
(10) Team reflexivity	4.97	0.77	0.23 **	0.29 **	0.33 **	0.25 **	0.30 **	0.34 **	0.43 **	0.49 **	0.69 **			
(11) Interdependence	5.39	0.79	0.19 **	0.24 **	0.35 **	0.35 **	0.36 **	0.36 **	0.39 **	0.58 **	0.47 **	0.40 **		
(12) Team cognitive diversity	4.33	0.93	−0.17 **	−0.25 **	−0.21 **	−0.21 **	−0.18 **	−0.24 **	−0.20 **	−0.25 **	−0.17 **	−0.16 **	−0.19 **	
(13) Team conflict	2.70	0.94	−0.23 **	−0.29 **	−0.39 **	−0.31 **	−0.29 **	−0.37 **	−0.40 **	−0.47 **	−0.28 **	−0.19 **	−0.27 **	0.39 **

N = 288 individuals; we used the four item SMM scale of Santos et al. (2015b) as second scale measure of SMMs to compare to the 5-PSMMS. **p < 0.01.

#### Aggregation

Our scale is intended for use at the team level, given its focus on the shared understanding of team members regarding different aspects of their work. As such, we conducted a further study using responses from teams with three or more members and aggregated the data of each variable by calculating average scores per team.

##### Participants

To assess whether aggregation would be permissible using our scale, we conducted a fourth study and recruited 340 new participants from 100 organizational teams with the help of research students. Of the participants, 46.8% were female and 25.0% identified as team leaders. The average age was 32.51 years (*SD* = 11.19). The average reported team size was 8.70 members (*SD* = 5.65) and on average we received 3.40 responses per team^4^. Participants who specified their industry predominantly worked in retail (34.3%), health care and social assistance (21.0%), finance and insurance (9.7%) and software (6.1%). Participants hailed from 25 countries, predominantly Germany (31.2%), the Netherlands (10.0%), Finland (5.9%), and Belgium (4.1%)^5^.

##### Measures

We used our 20 SMMs items.

##### Analyses

Aggregation can be justified if the *F*-test is significant as this indicates that variance between groups significantly exceeds variance within groups (Klein et al., 2000). Additionally, we calculated interrater reliability using the two intraclass correlation coefficients ICC1 and ICC2, where ICC2 values should be greater than ICC1 (Bliese, 2000). We also calculated *r*_*wg(j)*_ (the agreement between members within groups) where > 0.70 is deemed acceptable (James et al., 1993).

##### Results

Table 7 summarizes the data relating to aggregation. The *F*-test was significant at *p* < 0.001 for the whole SMM scale and all dimensions (except for composition, which was significant at *p* < 0.01). Aggregation is therefore justified for the whole scale and all five dimensions. The ICC1 value for the whole scale was 0.27 and values for the five dimensions ranged from 0.10 to 0.29. The ICC2 value for the whole scale was 0.62 and values for the five dimensions ranged from 0.32^6^ to 0.65. Fleiss (1986) considers ICC2 values < 0.40 to be poor, <0.75 to be fair to good and >0.75 to be excellent. Only the composition dimension had an ICC2 value below 0.40, hence the scale overall is acceptable. All ICC2 values exceeded their ICC1 counterparts, conforming to Bliese’s (2000) recommendations. Lastly, the *r*_*wg(j)*_ of the whole scale was 0.98 and ranged from 0.90 to 0.92 for the five SMM dimensions, meeting the requirement for aggregation (James et al., 1993). The scale is therefore suitable for measurement at the team level.

**TABLE 7:** Tests for data aggregation (Sample 4).

Dimension	*F*	MS between	MS within	ICC(1)	ICC(2)	Average *r*_*wg(j)*_
5-PSMMS	2.61 ***	1.17	0.45	0.27	0.62	0.98
Equipment	1.74 ***	1.40	0.81	0.14	0.42	0.91
Execution	2.84 ***	1.92	0.68	0.29	0.65	0.92
Interaction	2.35 ***	1.86	0.79	0.23	0.57	0.92
Composition	1.47 *	1.21	0.82	0.10	0.32	0.91
Temporal	1.99 ***	1.72	0.87	0.18	0.50	0.90

N_teams_ = 100; N_individuals_ = 340. *p < 0.05; ***p < 0.001.

## Discussion

Our aim was to consolidate the existing literature and from that create and validate a generally applicable SMM scale. To that end, we assessed more than 300 existing items and consolidated these divergent measurements from past research into a contemporary and utilitarian instrument that could be used to measure perceived SMMs across different tasks, teams, and organizational contexts. This was needed, because despite a large number of items and measures used in past research, there is currently no scale that incorporates team perceptions on all five core dimensions of SMMs. Past research, applying a plethora of measures, scales, and items – based on different dimensions – has provided in-depth insight into SMMs, yet has complicated synthesizing the literature, and this limits future research and the development of this field. Thus, we developed the 5-PSMMS and found support for its factor structure, convergent, discriminant and nomological validity, and aggregation to the team level. Specifically, the 5-PSMMS, with four items for each of the five dimensions, demonstrated psychometric adequacy over four separate datasets, containing 287, 275, 288, and 340 respondents, respectively.

### Theoretical and Methodological Implications

This study makes three important contributions to the SMM literature. First, the 5-PSMMS is the first and only scale that reflects all the core dimensions of SMMs currently used in literature. It provides researchers with an instrument that can be used to study and compare perceptions of SMMs, in a range of team or organization contexts. The scale shows five SMM dimensions (i.e., equipment, execution, interaction, composition, and temporal) and thus enables the study of perceived SMMs as a whole or in terms of these dimensions in isolation or combination with one another at the team level. In doing so we address the need for such an instrument which enables better research on perceived SMMs (e.g., Mohammed et al., 2010; Marhefka et al., 2020). The scale furthermore offers the potential for researchers to compare perceptions of SMMs with measures that assess SMMs’ structure and content (Marhefka et al., 2018, 2020). Although a generic scale sacrifices some depth, it has the benefit that misspecification of measurement (e.g., drawing on outdated or otherwise flawed task analyses) and misinterpretation (e.g., incorrectly interpreting keywords used by team members) of results are far less likely while offering the potential for comparison of results from research, not only within the field of SMMs, but also across disciplines (e.g., organizational learning, teamwork, or leadership). Hence, besides being used on its own, the 5-PSMMS also has the potential to serve as basis – or anchoring point – for the development of tailored instruments that can be applied in very specialized contexts (e.g., specialized medical teams performing unique operations) and enables the comparison between such different measures.

Second, we reflect upon the current common practice of studying SMMs in terms of only the task and/or team dimensions (e.g., Lim and Klein, 2006; Lee and Johnson, 2008) by critically evaluating the factor structure. In finding support for the five-factor model of SMMs, we support the original conceptualization of Cannon-Bowers et al. (1993) and incorporate the recently emerged temporal dimension as well (Mohammed et al., 2015; Santos et al., 2015a). As such, we advise that future research explicitly theorize why all five dimensions, or only some of them, are included in the particular study.

Third, our study incorporates *temporal* mental models into the general framework of SMMs in teams by highlighting that all five core dimensions are unique and should thus be acknowledged in future research. In addition, we also present important revisions to the temporal dimension of SMMs. Our deductive approach used items currently found in the literature and we discovered that, at present, the temporal dimension items include language explicitly linked to the execution dimension (e.g., Gevers et al., 2006; Mohammed et al., 2015; Santos et al., 2015a,^2016^; Marhefka et al., 2018). Hinkin (1998) advocates using items which measure only one issue, are clear in tone and structure, and do not lead to biased responses. Thus, the past conceptualization of temporal mental models is not appropriate as temporal factors can be relevant to all facets of a team and should thus not overly lean on one SMM dimension or another. Hence, our third contribution is that we challenge the current conceptualization, operationalization, and measurement of temporal mental models and offer an empirically supported refinement to this important, and burgeoning, aspect of SMMs.

### Practical Implications

We also make two important practical contributions. First, our new scale offers practitioners an accessible and user-friendly tool to identify potential issues involving SMMs in their teams. Alternative measures of SMMs often fall outside the ability of managers or consultants to apply, given their time-consuming nature and the limitation of not being applicable over a wide range of team and organizational contexts. The 20-item 5-PSMMS enables practitioners to identify whether teams have a shared understanding about their environment in general and allows for diagnosis of particular aspects of this environment by using one, or more, of the five sub-scales, which can be interpreted by computing teams’ mean score on each dimension (or the global SMM dimension) and comparing means across teams. As an example, a manager, consultant, or HR professional may find that a team does not share a perception on interaction mental models and can then address this by improving the communication style of team members. Given that we focused on *perceived* SMM, it would even be possible to see which (groups of) individuals would benefit most from an intervention, yet privacy concerns should be taken into account when doing so.

Second, we demonstrate the relevance of SMMs for a number of issues practitioners may encounter in teams. Our scale (conforming to theory) also related positively, but uniquely to important concepts like team learning, team reflexivity, and interdependence, and negatively to team cognitive diversity and intrateam conflict. By promoting the development and improvement of SMMs, it is likely that teams can avoid dysfunctional conflict (e.g., through the reduction of uncertainty about key issues in the team environment; Santos and Passos, 2013) and improve learning (e.g., through improved information sharing and understanding about which members possess which skills, knowledge, and abilities; Santos et al., 2016). Teams with more cognitively diverse members may also be liable to have less similar perceptions of SMMs (Schilpzand, 2010). Thus, managers working with cognitively diverse teams may improve team functioning by bridging differences in knowledge, values, or beliefs via the establishment of a shared understanding of the team’s environment.

Overall, through more widespread and repeated application of the 5-PSMMS, organizations can develop baseline assessments of SMMs in teams across the organization. In doing so they can identify teams that may benefit from intervention to develop a greater shared understanding about their environment and goals. This could enable targeted interventions to increase positive processes such as team learning and reflexivity, and reduce negative ones such as conflicts, and thus also improve performance.

### Strengths, Limitations, and Future Research

Akin to any other study, our research has some strengths as well as some limitations. Key amongst the strengths is our deductive approach following established scale development procedures (Hinkin, 1998; DeVellis, 2016), as this allowed us to critically assess and improve the current way of measuring within the SMM literature. Additionally, the use of four unique samples and the varied and detailed statistical tests are also strengths of the current research. A limitation is that our new scale refers to a perceived measure and not to a measure of SMM structure and context-dependent content (DeChurch and Mesmer-Magnus, 2010a). We cannot exclude the possibility that team members may sometimes mistakenly identify SMMs whereas, in reality, these may have drifted apart. For example, given that we found that, in a few teams, agreement amongst team members might be less than ideal for the composition dimension, we recommend researchers interested in that dimension to be especially diligent when they select their teams and respondents. Inaccurate SMMs pose a risk to team outcomes (Marhefka et al., 2018), however while actual SMMs may relay the structure of SMMs, this collectively represented structure may also be inaccurate (Marhefka et al., 2020). Therefore, our items and dimensions could be used in future research on their own, but could also offer complementary insights to measures of SMM structure, given the demonstrated outcomes of both SMM perception and structure (Marhefka et al., 2020).

In this paper, we followed an established methodology (Hinkin, 1998; DeVellis, 2016) to consolidate the measures of perceived SMMs into a scale which is theoretically more representative than past measures of perceived SMMs have been (cf. Johnson et al., 2007; Santos et al., 2015b), as we include all five core dimensions in one measure. Moreover, we also identified – and then corrected – a bias in how temporal mental models are conceptualized and measured in the contemporary literature. We then tested the nomological network and aggregation, to show that the 5-PSMMS is an interesting and appropriate tool for future researchers to use, and that it is suitable for use at the team level. One possible option would be to investigate the incremental and criterion related validity of our scale in more depth, as this was out of scope for our scale consolidation paper.

A related limitation is that our scale focused on generalized content of SMMs and thus loses some of the context-rich nature and thereby structure of SMMs captured, for instance, in card sorting or cognitive maps (DeChurch and Mesmer-Magnus, 2010a; Mohammed and Hamilton, 2012). However, these measures may also carry the risk of misspecification (Wildman et al., 2014), given that the structure of SMM is not immune to the risk of shared inaccurate representations among team members (Marhefka et al., 2020), and is very resource intensive to develop and use. For future research we advise that researchers consider why their studies require tailored measures such as card sorting or cognitive maps, given the difficulty involved in collecting large samples of data over time using such means. We also strongly advise to rigorously theorize and operationalize the five dimensions and measures, and suggest that our 5-PSMMS, as psychometrically adequate measure, can serve as a foundation, or benchmark, for doing so (Mohammed et al., 2010, 2017). *Post hoc* analyses indicated that neither nationality nor industry influenced results on the 5-PSMMS, supporting its robustness. Nonetheless, it is conceivable that nationality or industry could possibly influence perceived SMMs in some instances, and future research could thus also investigate the influence of such factors (cf. Niler et al., 2021). Overall, our scale allows for comparisons across a wide range of team and organizational contexts, permits easier longitudinal research, assists tailor-made studies, and facilitates the integration of research and practical findings across the SMM literature.

With regard to our data, although we used four unique datasets containing 1190 participants in total, the data was cross-sectional and self-reported. Such data is common in scale development papers (cf. Paulin and Griffin, 2017; Thomas and Lucas, 2019), yet our research design does much to allay these concerns as we draw on multiple samples, conduct multiple factor analyses, and actively screened responses based on a number of quality indications. Moreover, in our fourth dataset, the multilevel analyses go some way to address the risks posed by common method bias by aggregating the responses of multiple individuals to the team level. Future research could provide our scale to multiple sources (e.g., team members, leaders, HR specialists, etc.) and assess to what extent they agree about a team’s SMM. Additionally, future research could test our scale, its items and dimensions, over time by means of longitudinal research. Since our scale is generally applicable to a wide range of team and organizational contexts, such endeavors could be undertaken by different scholars, whilst still allowing their findings to quite easily be compared.

## Conclusion

Via our deductive scale consolidation approach, we were able to critically reflect upon the existing measures of SMM perceptions and dimensions in the contemporary literature and provide a new, theoretically and methodologically improved, scale that contains all five core dimensions. This allows researchers to more easily assess perceptions of SMMs in teams, and more easily compare findings across contexts, than is currently possible with custom-made, time- and resource intensive measures. Overall, our hope is thus that future research and practice on perceived SMMs can be done more rigorously, systematically, and easily with our new 20-item scale.

## Data Availability Statement

The datasets presented in this article are not readily available because the dataset cannot be shared due to privacy concerns of participants in the research. Requests to access the datasets should be directed to JJVR, j.jansenvanrensburg@maastrichtuniversity.nl.

## Ethics Statement

Ethical review and approval were not required for the study on human participants in accordance with the local legislation and institutional requirements. The patients/participants provided their written informed consent to participate in this study.

## Author Contributions

JJVR was the first author and responsible for all major elements of the manuscript including theory and literature, research design and execution, data collection, and results and discussion. CMS was a senior author and made significant contributions in literature and theory development, methods, results, and discussion of the results in addition to rigorous review of the manuscript. SBDJ was a senior author and made significant contributions in developing the literature and theory, methods, and discussion sections and in actively reviewing the manuscript. SU was last author and provided additional insight in the overall research design as well as literature and theory development. All the authors contributed to the article and approved the submitted version.

## Conflict of Interest

The authors declare that the research was conducted in the absence of any commercial or financial relationships that could be construed as a potential conflict of interest.

## Publisher’s Note

All claims expressed in this article are solely those of the authors and do not necessarily represent those of their affiliated organizations, or those of the publisher, the editors and the reviewers. Any product that may be evaluated in this article, or claim that may be made by its manufacturer, is not guaranteed or endorsed by the publisher.

## Footnotes

1 Similar to Anderson and Gerbing (1991), during the initial expert review, SMEs categorized items into different factors and associated dimensions in the first stage. We allowed SMEs to categorize each item under more than one dimension. As such we needed to adapt the calculation of Csv as the number of categorizations per item was allowed to be greater than the number of reviewers. Hence, the statistic was reformulated [*C_*sv*_* = *(n_*c*_ – n_*o*_)/N_*o*_*] where *n*_*c*_ is the number of times the item was categorized under the relevant dimension, *n*_*o*_ is the number of categorizations under other dimensions, and *N*_*o*_ is the total number of times the item was categorized under any dimension.

2
www.prolific.co

3 Our sample was relatively heterogeneous in terms of nationality and industry. Therefore, we conducted ANOVAs (with Tukey’s *post hoc* tests) to assess if the respondents’ mean scores on our 5-PSMMS measure and the seven nomological variables differed for each particular nationality or industry compared to one another. We focused on nationalities/industries for which we had at least two responses and thus included 21 nationalities and 26 industries in these analyses (the list of nationalities/industries can be provided by the first author upon request). Of the 4280 tests, only one was significant at *p* < 0.05 (i.e., the difference in means between the ‘Finance and Insurance’ and ‘Wholesale’ industries for the reflexivity scale). Overall, measures in the study were quite stable indicating the robustness of our study, yet we acknowledge that future research might want to study the effects of nationality and/or industry in more detail.

4 To assess possible effects of team tenure and the response rate, we conducted a correlation analysis testing tenure (*r* = –0.19, *p* = 0.22) and response rate (*r* = –0.14, *p* = 0.17). Since neither coefficient was significant, we conclude that tenure and response rate do not significantly affect the 5-PSMMS.

5 As per Sample 3, we conducted ANOVAs (with Tukey’s *post hoc* tests) to assess if the respondents’ mean scores on our 5-PSMMS measure differed for each particular nationality or industry compared to one another. We focused on nationalities/industries for which we had at least two responses and thus included 13 nationalities and 18 sectors in these analyses (the list of nationalities/industries can be provided by the first author upon request). Of the 231 tests, none were significant. Thus, as expected, our findings are robust, similar to what we reported for Sample 3 in footnote 3.

6 The ICC(2) value for composition (0.32) was just below the 0.40 cut-off value. Therefore, we reinvestigated the dataset which revealed that six of the 100 teams might contain a possible outlier which could skew the results. We reanalyzed the aggregation data for composition using the updated sample (*N_*teams*_* = 94, *N_*individuals*_* = 312) and found a sufficient ICC(2) value for the dimension (0.46). This indicates that for 94% of teams in our sample, the composition dimension was acceptable. We kept the 100 teams in our main findings, as all other statistics are adequate for all these teams.

## References

[B1] Anderson J. C. Gerbing D. W. (1991). Predicting the performance of measures in a confirmatory factor analysis with a pretest assessment of their substantive validities. *J. Appl. Psychol.* 76 732–740. 10.1037/0021-9010.76.5.732

[B2] Bachrach D. G. Lewis K. Kim Y. Patel P. C. Campion M. C. Thatcher S. M. B. (2019). Transactive memory systems in context: a meta-analytic examination of contextual factors in transactive memory systems development and team performance. *J. Appl. Psychol.* 104 464–493. 10.1037/apl0000329 30024196

[B3] Bliese P. D. (2000). “Within-group agreement, non-independence, and reliability: implications for data aggregation and analysis,” in *Multilevel Theory, Research, and Methods in Organizations: Foundations, Extensions, and New Directions*, eds Klein K. J. Kozlowski S. W. J. (Hoboken, NJ: Jossey-Bass), 349–381.

[B4] Burtscher M. J. Oostlander J. (2019). Perceived Mutual Understanding (PMU): development and initial testing of a German short scale for perceptual team cognition. *Eur. J. Psychol. Assess.* 35 98–108. 10.1027/1015-5759/a000360

[B5] Cannon-Bowers A. Salas E. Converse S. (1993). “Shared mental models in expert team decision making,” in *Individual & Group Decision Making*, ed. Castellan N. J. (Mahwah, NJ: Lawrence Erlbaum Associates), 221–245. 10.4324/9780203772744

[B6] Carley K. M. (1997). Extracting team mental models through textual analysis. *J. Organ. Behav.* 18 533–559. 10.1002/(SICI)1099-1379(199711)18:1+<533::AID-JOB906>3.0.CO;2-3

[B7] Carter S. M. West M. A. (1998). Reflexivity, effectiveness, and mental health in BBC-TV production teams. *Small Group Res.* 29, 583–601. 10.1177/1046496498295003

[B8] Colquitt J. A. Sabey T. B. Rodell J. B. Hill E. T. (2019). Content validation guidelines: evaluation criteria for definitional correspondence and definitional distinctiveness. *J. Appl. Psychol.* 104 1243–1265. 10.1037/apl0000406 30945879

[B9] de Wit F. R. Greer L. L. Jehn K. A. (2012). The paradox of intragroup conflict: a meta-analysis. *J. Appl. Psychol.* 97 360–390. 10.1037/a0024844 21842974

[B10] DeChurch L. A. Mesmer-Magnus J. R. (2010a). Measuring shared team mental models: a meta-analysis. *Group Dynamics: Theory Res. Practice* 14 1–14. 10.1037/a0017455

[B11] DeChurch L. A. Mesmer-Magnus J. R. (2010b). The cognitive underpinnings of effective teamwork: a meta-analysis. *J. Appl. Psychol.* 95 32–53. 10.1037/a0017328 20085405

[B12] DeVellis R. F. (2016). *Scale Development: Theory and Applications.* Newcastle upon Tyne: SAGE Publications.

[B13] Edmondson A. (1999). Psychological safety and learning behavior in work teams. *Admin. Sci. Quar.* 44, 350–383. 10.2307/2666999

[B14] Edwards B. D. Day E. A. Arthur W. Bell S. T. (2006). Relationships among team ability composition, team mental models, and team performance. *J. Appl. Psychol.* 91, 727–736. 10.1037/0021-9010.91.3.727 16737368

[B15] Ellwart T. Konradt U. Rack O. (2014). Team mental models of expertise location: validation of a field survey measure. *Small Group Res.* 45 119–153. 10.1177/1046496414521303

[B16] Fleiss J. (1986). *The Design and Analysis of Clinical Experiments.* Hoboken, NJ: John Wiley and Sons. 10.1002/9781118032923

[B17] Gevers J. M. Rutte C. G. Van Eerde W. (2006). Meeting deadlines in work groups: implicit and explicit mechanisms. *Appl. Psychol. Inter. Rev.* 55 52–72. 10.1111/j.1464-0597.2006.00228.x

[B18] Greer L. L. Jehn K. A. Mannix E. A. (2008). Conflict transformation: a longitudinal investigation of the relationships between different types of intragroup conflict and the moderating role of conflict resolution. *Small Group Res.* 39 278–302. 10.1177/1046496408317793

[B19] Guchait P. Hamilton K. Hua N. (2014). Personality predictors of team taskwork understanding and transactive memory systems in service management teams. *Int. J. Contemporary Hospitality Manag.* 26 401–425. 10.1108/IJCHM-05-2013-0197

[B20] Gurtner A. Tschan F. Semmer N. K. Nägele C. (2007). Getting groups to develop good strategies: effects of reflexivity interventions on team process, team performance, and shared mental models. *Organ. Behav. Hum. Decision Proc.* 102 127–142. 10.1016/j.obhdp.2006.05.002

[B21] Hair J. F. Jr. Black W. C. Babin B. J. Anderson R. E. (2018). *Multivariate Data Analysis*, 8th Edn. Boston, MA: Cengage Learning.

[B22] Hardesty D. M. Bearden W. O. (2004). The use of expert judges in scale development: implications for improving face validity of measures of unobservable constructs. *J. Bus. Res.* 57 98–107. 10.1016/s0148-2963(01)00295-8

[B23] Hinkin T. R. (1998). A brief tutorial on the development of measures for use in survey questionnaires. *Organ. Res. Methods* 1 104–121. 10.1177/109442819800100106

[B24] Hsu J. S. Chang J. Y. Klein G. Jiang J. J. (2011). Exploring the impact of team mental models on information utilization and project performance in system development. *Int. J. Project Manag.* 29 1–12. 10.1016/j.ijproman.2009.12.001

[B25] James L. R. Demaree R. J. Wolf G. (1993). rwg: an assessment of within group interrater agreement. *J. Appl. Psychol.* 78 306–309. 10.1037/0021-9010.78.2.306

[B26] Jehn K. (1995). A multimethod examination of the benefits and detriments of intragroup conflict. *Administrative Sci. Quarterly* 40 256–282. 10.2307/2393638

[B27] Jehn K. A. Mannix E. A. (2001). The dynamic nature of conflict: a longitudinal study of intragroup conflict and group performance. *Acad. Manag. J.* 44 238–251. 10.2307/3069453

[B28] Johnson T. E. Lee Y. Lee M. O’Connor D. L. Khalil M. K. Huang X. (2007). Measuring sharedness of team-related knowledge: design and validation of a shared mental model instrument. *Hum. Resource Dev. Int.* 10 437–454. 10.1080/13678860701723802

[B29] Kang H. R. Yang H. D. Rowley C. (2006). Factors in team effectiveness: cognitive and demographic similarities of software development team members. *Hum. Relations* 59 1681–1710. 10.1177/0018726706072891

[B30] Klein K. J. Bliese P. D. Kozlowski S. W. J. Dansereau F. Gavin M. B. Griffin M. A. (2000). “Multilevel analytical techniques: commonalities, differences, and continuing questions,” in *Multilevel Theory, Research, and Methods in Organizations: Foundations, Extensions, and New Directions*, eds Klein K. J. Kozlowski S. W. J. (Hoboken, NJ: Jossey-Bass), 512–553.

[B31] Klimoski R. Mohammed S. (1994). Team mental model: construct or metaphor? *J. Manag.* 20 403–437. 10.1016/0149-2063(94)90021-3

[B32] Kniffin K. M. Narayanan J. Anseel F. Antonakis J. Ashford S. P. Bakker A. B. (2021). COVID-19 and the workplace: implications, issues, and insights for future research and action. *Am Psychol.* 76 63–77. 10.1037/amp0000716 32772537

[B33] Kozlowski S. W. J. Bell B. S. (2013). “Work groups and teams in organizations: review update,” in *Handbook of Psychology: Industrial and Organizational Psychology*, Vol. 12, 2nd Edn. eds Schmitt N. Highhouse S. (Hoboken, NJ: Wiley), 412–469.

[B34] Lee M. Johnson T. E. (2008). Understanding the effects of team cognition associated with complex engineering tasks: dynamics of shared mental models, task-SMM, and team-SMM. *Perform. Improvement Quarterly* 21 73–95. 10.1002/piq.20032

[B35] Levesque L. L. Wilson J. M. Wholey D. R. (2001). Cognitive divergence and shared mental models in software development project teams. *J. Organ. Behav.* 22 135–144. 10.1002/job.87

[B36] Lewis K. (2003). Measuring transactive memory systems in the field: scale development and validation. *J. Appl. Psychol.* 88 587–604. 10.1037/0021-9010.88.4.587 12940401

[B37] Lim B.-C. Klein K. J. (2006). Team mental models and team performance: a field study of the effects of team mental model similarity and accuracy. *J. Organ. Behav.* 27 403–418. 10.1002/job.387

[B38] Marhefka J. Mohammed S. Hamilton K. Tesler R. Mancuso V. McNeese M. (2018). We are not in sync, but we think we are: actual versus perceived temporal team mental models. *J. Organ. Psychol.* 18 79–93. 10.33423/jop.v18i4.87

[B39] Marhefka J. Mohammed S. Hamilton K. Tester R. Mancuso V. McNeese M. D. (2020). “Mismatches between perceiving and actually sharing temporal mental models: implications for distributed teams,” in *Foundations and Theoretical Perspectives of Distributed Team Cognition*, eds McNeese M. D. Salas E. Endsley M. R. (Boca Raton, FL: CRC Press), 159–183. 10.1201/9780429459795-7

[B40] Marks M. A. Mathieu J. E. Zaccaro S. J. (2001). A temporally based framework and taxonomy of team processes. *Acad. Manag. Rev.* 26 356–376. 10.2307/259182

[B41] Marks M. A. Zaccaro S. J. Mathieu J. E. (2000). Performance implications of leader briefings and team-interaction training for team adaptation to novel environments. *J. Appl. Psychol.* 85, 971–986. 10.1037/0021-9010.85.6.971 11125660

[B42] Martins L. L. Schilpzand M. C. Kirkman B. L. Ivanaj S. Ivanaj V. (2013). A contingency view of the effects of cognitive diversity on team performance: the moderating roles of team psychological safety and relationship conflict. *Small Group Res.* 44 96–126. 10.1177/1046496412466921

[B43] Mathieu J. E. Gallagher P. T. Domingo M. A. Klock E. A. (2019). Embracing complexity: reviewing the past decade of team effectiveness research. *Ann. Rev. Organ. Psychol. Organ. Behav.* 6 17–46. 10.1097/MED.0000000000000549 32618635 PMC7596883

[B44] Mathieu J. E. Heffner T. S. Goodwin G. F. Cannon-Bowers J. A. Salas E. (2005). Scaling the quality of teammates’ mental models: equifinality and normative comparisons. *J. Organ. Behav.* 26 37–56. 10.1002/job.296

[B45] Mathieu J. E. Heffner T. S. Goodwin G. F. Salas E. Cannon-Bowers J. A. (2000). The influence of shared mental models on team process and performance. *J. Appl. Psychol.* 85 273–283. 10.1037/0021-9010.85.2.273 10783543

[B46] Maynard M. T. Gilson L. L. (2014). The role of shared mental model development in understanding virtual team effectiveness. *Group Organ. Manag.* 39, 3–32. 10.1177/1059601113475361

[B47] Mesmer-Magnus J. Niler A. A. Plummer G. Larson L. E. DeChurch L. A. (2017). The cognitive underpinnings of effective teamwork: a continuation. *Career Dev. Int.* 22 507–519. 10.1108/CDI-08-2017-0140

[B48] Millward L. J. Jeffries N. (2001). The team survey: a tool for health care team development. *J. Adv. Nurs.* 35 276–287. 10.1046/j.1365-2648.2001.01844.x 11442706

[B49] Mohammed S. Hamilton K. (2012). “Studying team cognition: the good, the bad, and the practical,” in *Research Methods for Studying Groups and Teams: A Guide to Approaches, Tools, and Technologies*, eds Hollingshead A. B. Poole M. S. (Milton Park: Routledge).

[B50] Mohammed S. Harrison D. A. (2013). The clocks that time us are not the same: a theory of temporal diversity, task characteristics, and performance in teams. *Organ. Behav. Hum. Decision Proc.* 122 244–256. 10.1016/j.obhdp.2013.08.004

[B51] Mohammed S. Ferzandi L. Hamilton K. (2010). Metaphor no more: a 15-year review of the team mental model construct. *J. Manag.* 36 876–910. 10.1177/0149206309356804

[B52] Mohammed S. Hamilton K. Sánchez-Manzanares M. Rico R. (2017). “Team cognition,” in *The Wiley Blackwell Handbook of the Psychology of Team Working and Collaborative Processes*, eds Salas E. Rico R. Passmore J. (Hoboken, NJ: John Wiley & Sons), 369–392. 10.1002/9781118909997

[B53] Mohammed S. Hamilton K. Tesler R. Mancuso V. McNeese M. (2015). Time for temporal team mental models: expanding beyond “what” and “how” to incorporate “when”. *Eur. J. Work Organ. Psychol.* 24 693–709. 10.1080/1359432x.2015.1024664

[B54] Mohammed S. Klimoski R. Rentsch J. R. (2000). The measurement of team mental models: we have no shared schema. *Organ. Res. Methods* 3 123–165. 10.1177/109442810032001

[B55] Mohammed S. Rico R. Alipour K. (2021). Team cognition at a crossroad: toward conceptual integration and network configurations. *Acad. Manag. Ann.* 15 455–501. 10.5465/annals.2018.0159

[B56] Mohammed S. Tesler R. Hamilton K. (2012). “Time and team cognition: toward greater integration of temporal dynamics,” in *Theories of Team Cognition: Cross-disciplinary Perspectives*, eds Salas E. Fiore S. M. Letsky M. P. (Milton Park: Routledge). 10.5465/annals.2018.0159

[B57] Niler A. A. Mesmer-Magnus J. R. Larson L. E. Plummer G. DeChurch L. A. Contractor N. S. (2021). Conditioning team cognition: a meta-analysis. *Organ. Psychol. Rev.* 11 144–174. 10.1177/2041386620972112

[B58] Paulin D. Griffin B. (2017). Team incivility climate scale: development and validation of the team-level incivility climate construct. *Group Organ. Manag.* 42 315–345. 10.1177/1059601115622100

[B59] Polzer J. T. Crisp C. B. Jarvenpaa S. L. Kim J. W. (2006). Extending the faultline model to geographically dispersed teams: how colocated subgroups can impair group functioning. *Acad. Manag. J.* 49 679–692. 10.2307/20159792

[B60] Rentsch J. R. Delise L. A. Hutchison S. (2009). “Team member schema accuracy and team member schema congruence: in search of the team mindmeld,” in *Team effectiveness in complex organizations*, eds Salas E. Goodwin G. F. Burke C. S. (Milton Park: Routledge).

[B61] Roe R. A. (2008). Time in applied psychology: the study of “what happens” rather than “what is”. *Eur. Psychol.* 13 37–52. 10.1027/1016-9040.13.1.37

[B62] Santos C. M. Passos A. M. (2013). Team mental models, relationship conflict and effectiveness over time. *Team Perform. Manag.* 19 363–385. 10.1108/TPM-01-2013-0003

[B63] Santos C. M. Passos A. M. Uitdewilligen S. (2016). When shared cognition leads to closed minds: temporal mental models, team learning, adaptation and performance. *Eur. Manag. J.* 34 258–268. 10.1016/j.emj.2015.11.006

[B64] Santos C. M. Uitdewilligen S. Passos A. M. (2015a). A temporal common ground for learning: the moderating effect of shared mental models on the relation between team learning behaviours and performance improvement. *Eur. J. Work Organ. Psychol.* 24 710–725. 10.1080/1359432X.2015.1049158

[B65] Santos C. M. Uitdewilligen S. Passos A. M. (2015b). Why is your team more creative than mine? the influence of shared mental models on intra-group conflict, team creativity and effectiveness. *Creat. Innovation Manag.* 24 645–658. 10.1111/caim.12129

[B66] Santos C. M. Uitdewilligen S. Passos A. M. Marques-Quinteiro P. Maynard M. T. (2021). The effect of a concept mapping intervention on shared cognition and adaptive team performance over time. *Group Organ. Manag.* 46, 984–1026. 10.1177/1059601120981623

[B67] Schilpzand M. C. (2010). *Cognitive Diversity and Team Performance: The Roles of Team Mental Models and Information Processing Mechanisms.* Doctoral dissertation, Georgia: SMARTech.

[B68] Schippers M. C. West M. A. Dawson J. F. (2015). Team reflexivity and innovation: the moderating role of team context. *J. Manag.* 41 769–588. 10.1177/0149206312441210

[B69] Smith-Jentsch K. A. Campbell G. E. Milanovich D. M. Reynolds A. M. (2001). Measuring teamwork mental models to support training needs assessment, development, and evaluation. *J. Organ. Behav.* 22 179–194. 10.1002/job.88

[B70] Smith-Jentsch K. A. Cannon-Bowers J. A. Tannenbaum S. I. Salas E. (2008). Guided team self-correction: impacts on team mental models, processes, and effectiveness. *Small Group Res.* 39 303–327. 10.1177/1046496408317794

[B71] Staples D. S. Webster J. (2008). Exploring the effects of trust, task interdependence and virtualness on knowledge sharing in teams. *Inform. Systems J.* 18 617–640. 10.1111/j.1365-2575.2007.00244.x

[B72] Tesler R. Mohammed S. Hamilton K. Mancuso V. McNeese M. (2018). Mirror, mirror: guided storytelling and team reflexivity’s influence on team mental models. *Small Group Res.* 49 267–305. 10.1177/1046496417722025

[B73] Thomas B. Lucas K. (2019). Development and validation of the workplace dignity scale. *Group Organ. Manag.* 44 72–111. 10.1177/1059601118807784

[B74] Uitdewilligen S. Rico R. Waller M. J. (2018). Fluid and stable: dynamics of team action patterns and adaptive outcomes. *J. Organ. Behav.* 39 1113–1128. 10.1002/job.2267

[B75] Uitdewilligen S. Waller M. J. Pitariu A. H. (2013). Mental model updating and team adaptation. *Small Group Res.* 44 127–158. 10.1177/1046496413478205

[B76] Uitdewilligen S. Waller M. J. Roe R. A. Bollen P. (2021). The effects of team mental model complexity on team information search and performance trajectories. *Group Organ. Manag.* 10.1177/10596011211023219 [Epub ahead of print].

[B77] Van den Bossche P. Gijselaers W. Segers M. Woltjer G. Kirschner P. (2011). Team learning: building shared mental models. *Instruct. Sci.* 39 283–301. 10.1007/s11251-010-9128-3

[B78] Van den Bossche P. Gijselaers W. H. Segers M. Kirschner P. A. (2006). Social and cognitive factors driving teamwork in collaborative learning environments: team learning beliefs and behaviors. *Small Group Res.* 37, 490–521. 10.1177/1046496406292938

[B79] Van der Vegt G. S. Janssen O. (2003). Joint impact of interdependence and group diversity on innovation. *J. Manag.* 29 729–751. 10.1016/S0149-2063_03_00033-3

[B80] Van der Vegt G. S. Van de Vliert E. (2005). Effects of perceived skill dissimilarity and task interdependence on helping in work teams. *J. Manag.* 31 73–89. 10.1177/0149206304271382

[B81] Van der Vegt G. S. De Jong S. B. Bunderson J. S. Molleman E. (2010). Power asymmetry and learning in teams: the moderating role of performance feedback. *Organ. Sci.* 21 347–361. 10.1287/orsc.1090.0452 19642375

[B82] Waller M. J. Gupta N. Giambatista R. C. (2004). Effects of adaptive behaviors and shared mental models on control crew performance. *Manag. Sci.* 50 1534–1544. 10.1287/mnsc.1040.0210 19642375

[B83] West M. A. (1996). “Reflexivity and work group effectiveness: a conceptual integration,” in *Handbook of Work Group Psychology*, ed. West M. A. (Hoboken, NJ: Wiley), 555–579. 10.1080/00223980.2015.1050977

[B84] Wildman J. L. Salas E. Scott C. P. R. (2014). Measuring cognition in teams: a cross-domain review. *Hum. Factors* 56 911–941. 10.1177/0018720813515907 25141596

[B85] Worthington R. L. Whittaker T. A. (2006). Scale development research: a content analysis and recommendations for best practices. *Counsel. Psychol.* 34, 806–838. 10.1177/0011000006288127

